# Associations Between Vitamin D Levels and Risk of Heart Failure: A Bidirectional Mendelian Randomization Study

**DOI:** 10.3389/fnut.2022.910949

**Published:** 2022-05-19

**Authors:** Ning Gao, Xuebiao Li, Minjian Kong, Ming Ni, Dongdong Wei, Xian Zhu, Yifan Wang, Ze Hong, Aiqiang Dong

**Affiliations:** Department of Cardiovascular Surgery, The Second Affiliated Hospital of Zhejiang University School of Medicine, Hangzhou, China

**Keywords:** vitamin D, heart failure, Mendelian randomization, the causal link, genome-wide association study

## Abstract

**Background:**

Although studies suggest that concentrations of serum 25-hydroxyvitamin D (25(OH)D) are lower in individuals with Heart Failure (HF), the beneficial effects of vitamin D supplementation are controversial. Therefore, in this study, we aimed to determine whether there is a causal relationship between serum Vitamin D (VD) levels and HF.

**Methods:**

We obtained genetic instruments from the largest available genome-wide association study (GWAS) of European descent for 25(OH)D (443, 734 individuals) to investigate the association with HF (47,309 cases, 930,014 controls), and vice versa. Two-sample bidirectional Mendelian Randomization (MR) analysis was performed to infer the causality. In addition to the primary analysis using inverse variance-weighted (IVW) MR, we applied five additional methods to control for pleiotropy [MR-Egger, weighted median, Maximum-likelihood, MR-robust adjusted profile score (MR-RAPS) and MR-pleiotropy residual sum and outlier (MR-PRESSO)] and compared their respective MR estimates. We also performed a sensitivity analysis to ensure that our results were robust.

**Results:**

Mendelian randomized analysis showed that increased serum 25(OH)D was associated with a lower risk of HF in the IVW method (odds ratio [OR] = 0. 81;95%CI, 0.70–0.94, *P* = 0.006). In the reverse MR analyses, the genetic predisposition to HF was negatively correlated with serum 25(OH)D level (OR = 0. 89;95%CI, (0.82–0.97), *P* = 0.009).

**Conclusion:**

Our study revealed the possible causal role of 25(OH)D on decreasing the risk for HF. Meanwhile, reverse MR analysis suggested that HF may be associated with lower vitamin D levels, it could be the potential implications for dietary recommendations.

## Introduction

Heart failure (HF) is a complex clinical syndrome, which often occurs in ventricular filling or impaired blood ejection due to cardiac dysfunction (1). Heart failure harasses about 64.3 million people globally (1, 2), and its prevalence is rising (3). A large meta-analysis showed that the 1-, 2-, 5-, and 10-year survival rates of HF patients were 87, 73, 57, and 35%, respectively, which undoubtedly caused a huge social and medical burden (4). The interaction of genetic and environmental factors were suggested as a potential mechanism of HF (5–8), and additional nutritional support may be an important factor (9).

Vitamin D is a nutrient obtained from sunlight, dietary and supplemental intake. Vitamin D supplementation improves hypertension (10) and reduces the risk of myocardial infarction (11), whereas vitamin D deficiency appears to increase the risk of HF (12). Vitamin D has a pleiotropic role in heart failure pathology (13). However, controversy has been unsettled with regard to the true link between 25(OH)D and HF. Cohort studies showed that vitamin D supplementation reduced in-hospital mortality in heart failure patients (14). Similarly, a prospective cohort study of 19,092 individuals showed that patients with vitamin D deficiency had a higher risk (HR: 1.61, 95%CI: 1.06–2.43) of hospitalization for HF than patients with normal levels (15). Similarly, a prospective cohort study of 19,092 people showed that patients with vitamin D deficiency had a higher risk of hospitalization (HR: 1.61, 95%CI: 1.06–2.43) (15). However, a recent large randomized controlled trial (RCT) of vitamin D supplementation reported futility results (16). Notably, the previously observed associations might be confounded by the relatively small sample size and confounding factors. RCTs also had some problems. Baseline 25(OH)D concentrations were not considered for included subjects and the trial follow-up period was limited, so subgroups with low 25(OH)D concentrations could not be effectively tested (16).

As an emerging method of epidemiological causal inference, Mendelian randomization (MR) has achieved great success in finding potential therapeutic targets for disease. MR relies on the natural random classification of genetic variation during meiosis, resulting in a grouping process similar to RCT in the population (17). Because external interference usually does not change the germline DNA sequence (18), it can be said that MR is not easy to reverse causality (19). More importantly, genetic variants are typically unrelated to confounders, so MR provides a more reliable understanding of the potential causal relationship between exposure and outcome (20). Finally, because genetic variation remains stable throughout life, MR research provides insight from a lifetime of genetically altered risk factors. Serum 25-hydroxyvitamin D (25(OH)D) reflects the combined effects of supplementation and skin production, so the level of serum 25 (OH)D is considered to be the standard measure of vitamin D status in subjects (21). Here, the bidirectional MR was conducted to examine for the first time if there is a causal association between vitamin D and HF risk.

## Materials and Methods

### Data Sources and Study Design

To reduce population stratification bias, all datasets were from European Caucasian ancestry. Table 1 shows detailed information about the data source. Summary statistic data for 25(OH)D were derived from a large meta-analysis of GWAS (22), consisting of 401,460 Europeans from the UK Biobank (UKB) and 42,274 Europeans from an international consortium of adult individuals of European ancestry. And the GWAS summary statistics of HF traits were from the Heart Failure Molecular Epidemiology for Therapeutic Targets (HERMES) consortium (23), including 47,309 cases and 930,014 controls from 26 studies (17 cohort studies and 9 case-control studies). Details of this study were provided in the Supplementary Table 1.

**Table 1:** Characteristics of data sources and strength of IVs used in the Mendelian randomization study.

**Traits**	**Data sources**	**Sample size**	**Ancestry**	***F*-statistic (total)**
25-hydroxy vitamin D	UK Biobank et al.	443,734	European	11.62
Heart failure	HERMES	47,309/930,014	European	16.72

*HERMES, Heart Failure Molecular Epidemiology for Therapeutic Targets; F = R^2^(N-K-1)/[K(1-R^2^)], R^2^ = 2 × (1-EAF) × EAF × (β/SD)^2^, SD = SE × N^1/2^, where EAF is the effect allele frequency, β is the estimated effect on adipokines, N is the sample size of the GWAS and SE is the standard error of the estimated effect*.

**Figure 1:**
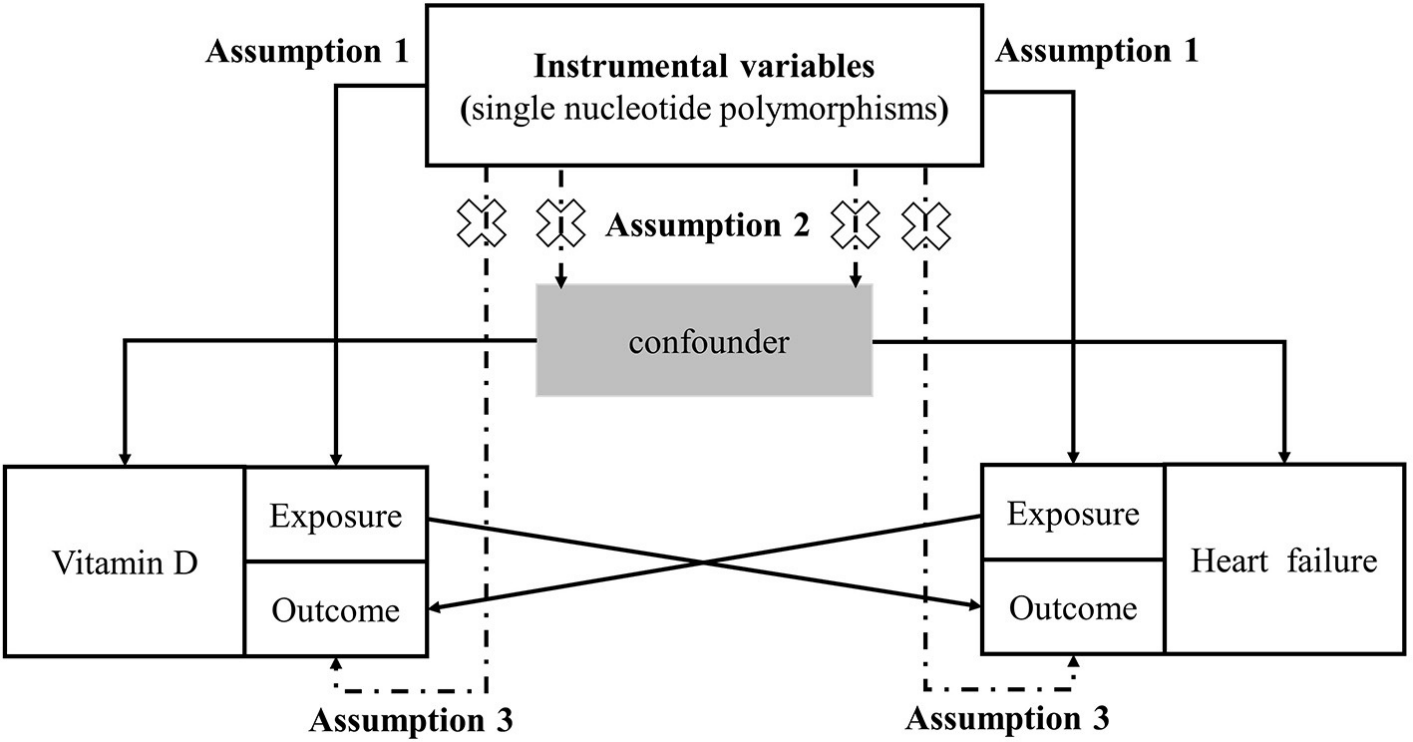
Study design flowchart of the Mendelian randomization study. The Mendelian randomization method is based on three hypotheses: (1) the instrumental variables is closely related to exposure; (2) instrumental variables is independent of any confounding factor; (3) instrumental variables affects the results only through exposure but not through other ways.

We used the two-sample bidirectional MR analysis, evaluating the causal association between vitamin D and heart failure (24). The MR analysis should be carried out under three basic assumptions: (1) The genetic variants are closely associated with exposure; (2) the genetic variants are not associated with any potential confounders; (3) the genetic variants are not associated with outcome except via the way of exposure (25) (Figure 1). All the original studies have obtained ethical approval and informed consent. This study was reported in accordance with the latest Strengthening the Reporting of Observational Studies in Epidemiology Using Mendelian Randomization (STROBE-MR) guideline (26).

### Phenotype Data

Vitamin D. Serum levels of 25-hydroxyvitamin D (nmol/L) were measured using the Diasorin assay (a chemiluminescent immunoassay method) with a detection range of 10 nmol/L <25(OH)D <375 nmol/L. Measurements were performed at baseline (2006–2010) and/or the first follow-up visit (2012–2013). Participants were stratified by the concentration of serum 25(OH)D levels (adequate [>75 nmol/L], sufficient [50–75 nmol/L], insufficient [25–49 nmol/L], and deficient [<25 nmol/L]) (22).

Heart failure. Cases included participants with a clinical diagnosis of HF of any etiology with no inclusion criteria based on left ventricle (LV) ejection fraction; controls were participants without HF. Definitions used to adjudicate HF status within each study are detailed in the Supplementary Table 1 and baseline characteristics for each study are provided in Supplementary Table 2.

### Selection of IVs

All genetic variants significantly associated with 25(OH)D (*P* < 5 × 10^−8^) were selected as IVs. The corresponding linkage disequilibrium was tested to confirm that there were any SNPs in a linkage disequilibrium state and the SNPs were independent by pruning SNPs within a 10,000 kb window with an *r*^2^ < 0.001 threshold. We used a conservative analysis model to remove SNPs related to confounding factors in MR analysis (25). In order to exclude the potential pleiotropy effect, we searched the secondary phenotype of each SNP in PhenoScanner V2 (27), assessing whether SNPs were potentially associated with confounders or risk factors for outcomes. The SNPs corresponding to the phenotype related to the results was excluded, and the remaining SNPs were used as the IVs for clear causal inference. Specifically, when HF was the outcome, we excluded SNPs associated with hypertension and myocardial infarction. Likewise, in the inverse MR analysis, we excluded SNPs associated with 25(OH)D levels: basal metabolic rate.

To avoid potential weak instrumental bias, Variance (*R*^2^) and *F* statistics were used to assess the strength of IVs (28, 29). We adopted the latest and most stringent calculation method. *F* = *R*^2^ (*N*-*K*-1)/[*K*(1-*R*^2^)]. In this equation, *R*^2^ refers to the cumulative explained variance of the selected SNP during exposure, *K* is the number of SNP for the final analysis, and *N* is the number of samples of the selected GWAS. If *F* > 10, the correlation between the IVs and exposure is considered strong enough, and the results of the MR analysis can avoid being affected by weak tool bias.

### Mendelian Randomization Analyses

We harmonized the aggregated SNP-25 (OH) D and SNP-HF statistics to ensure that the alleles of each SNP were consistent between exposure and results. In MR analysis, the inverse variance weighting (IVW) method of different models was used as the main analysis method according to heterogeneity (19). At the same time, median weighting (30), MR-Egger (31), Maximum-likelihood (32), MR-robust adjusted profile score (MR-RAPS) (33) and MR-pleiotropy residual sum and outlier (MR-PRESSO) (34) are used to infer the causal relationship. Each method makes different assumptions about the effectiveness of the IVs. Median weighting can be estimated when the 50%IVs are invalid (30). Although the statistical ability of the MR-Egger method is low, it provides an estimate after correcting the multiple effects (31). MR-RAPS corrects horizontal multiplicity by using robust adjusted contour scores, which reduces the deviation caused by horizontal multiplicity (33). MR-PRESO method can automatically detect outliers in IVW linear regression and automatically remove outliers to provide corrected MR estimation (34). In a word, we used all these methods to study causality comprehensively. Details of the analysis method can be found in Supplementary Material.

### Sensitivity Analyses

Various methods were introduced into this study for sensitivity analysis. First, Cochran's Q test was applied to assess heterogeneity between individual genetic variants estimates. If the *p*-value was less than 0.05, the final results of MR were referred to a random-effects model of IVW, otherwise, a fixed-effects model was used. Second, we used the MR-Egger Intercept method to test the horizontal pleiotropy of IVs (31). In MR-Egger test, the intercept estimates the average pleiotropic effect across the genetic variants, and if the *p*-value is less than 0.05, it indicates that IVW estimates might be biased. Third, we conducted the leave-one-out sensitivity test to examine whether the results were caused by any single SNP. Fourth, funnel plots and forest plots were generated to directly detect the existence of pleiotropy.

All statistical analyses were performed using the “TwoSampleMR,” “MR-PRESSO,” and “mr.raps” packages in R software, Version 4.1.2. All *P*-values were two-sided, and *P* < 0.05 was deemed as suggestive significance.

## Results

### Characteristics of the Selected SNPs

Population details for 25(OH)DGWAS are presented in the Supplementary Table 3. We extracted IVs significantly associated (*P* < 5 × 10^−8^) with 25(OH)D from the GWAS (22) and removed linkage disequilibrium (*r*^2^ < 0.001, 10,000-kb). SNPs associated with hypertension (rs75865451, rs62493791, rs951914, rs2229742, rs148747986) and myocardial infarction (rs12798050) were retrieved from the PhenoScanner database, which were excluded as risk factors for HF. Meanwhile, we removed the SNPs for being palindromic with intermediate allele frequencies. In the end, 137 SNPs were included in further analysis (Supplementary Table 4). In the test of IVs strength, no evidence of weak tool bias was found (*F*-statistic > 10) (Table 1).

For reverse MR analysis, there were 12 significantly related SNPs after removing LD. Using the same method, three SNP related to the basal metabolic rate (rs4135240, rs4766578, rs56094641) in the PhenoScanner database were excluded. At the same time, we removed the SNPs for being palindromic. A total of 6 SNPs were screened for subsequent reverse MR analysis, and these SNPs were unlikely to be subject to weak IVs bias (*F*-statistic > 10) (Table 1 and Supplementary Table 5).

### Association of Genetically Predicted 25(OH)D Levels With Risk of HF

As shown in Table 2, the IVW method showed a causal relationship between the increase in genetically predicted 25(OH)D levels and the reduced the risk of heart failure (OR = 0.81;95%CI, 0.70–0.94, *P* = 0.006). The Maximum likelihood method, MR-PRESSO, MR-RAPS analysis were consistent with IVW method (Supplementary Figure 1). The causal estimation of the MR-PRESSO method before and after outlier correction is consistent, which shows that the result of MR analysis is reliable.

**Table 2:** Mendelian randomization of circulating 25-hydroxyvitamin D levels and the risk of Heart failure.

**Exposure**	**Outcome**	**MR method**	**SNP (*n*)**	**β (95% CI)**	**OR (95% CI)**	***P***
25(OH)D	HF	MR-Egger	137	−0.32 (−0.67 to −0.03)	0.73 (0.51–1.03)	0.08
		Weighted median	137	−0.16 (−0.38 to 0.06)	0.85 (0.68–1.06)	0.15
		IVW (random effects)	137	−0.21 (−0.36 to −0.06)	0.81 (0.70–0.94)	**0.006**
		Maximum likelihood	137	−0.21 (−0.34 to −0.09)	0.81 (0.72–0.91)	**0.001**
		IVW (fixed effects)	137	−0.21 (−0.33 to −0.09)	0.81 (0.72–0.91)	**<0.001**
		MR–PRESSO (Raw)	137	−0.22 (−0.36 to −0.08)	0.81 (0.70–0.93)	**0.003**
		MR-PRESSO (Outlier-corrected)	137	−0.15 (−0.27 to −0.03)	0.86 (0.76–0.97)	**0.02**
		MR-RAPS	137	−0.18 (−0.30 to −0.05)	0.84 (0.74–0.95)	**0.005**

*SNPs, Single nucleotide polymorphisms; OR, Odds ratio; CI, Confidence interval; IVW, inverse-variance weighted; MR-RAPS, MR-robust adjusted profile score; MR-PRESSO, MR-pleiotropy residual sum and outlier; 25(OH)D, 25-hydroxyvitamin D; HF, Heart failure*.

*Numbers in bold are statistically significant*.

### Association of Genetically Predicted HF With 25(OH)D Levels

Table 3 showed the results of reverse MR analysis, showing a negative correlation between heart failure and serum 25(OH)D levels based on the IVW method [OR = 0.89;95%CI, (0.82–0.97), *P* = 0.009]. At the same time, the median weighting method, Maximum likelihood method, MR-PRESSO and MR-RAPS method (Supplementary Figure 2) were consistent with IVW method. The MR-PRESSO-raw estimation was consistent with the MR-PRESSO-Outlier-corrected, which proved the reliability of the reverse MR analysis results.

**Table 3:** Mendelian randomization of the risk of Heart failure and circulating 25-hydroxyvitamin D levels.

**Exposure**	**Outcome**	**MR method**	**SNP (n)**	**β (95% CI)**	**OR (95% CI)**	***P***
HF	25(OH)D	MR-Egger	6	0.05 (−0.16 to −0.26)	1.05 (0.85 to 1.30)	0.66
		Weighted median	6	−0.06 (−0.11 to −0.02)	0.94 (0.90 to 0.98)	**0.008**
		IVW (random effects)	6	−0.11 (−0.20 to −0.03)	0.89 (0.82 to 0.97)	**0.009**
		Maximum likelihood	6	−0.13 (−0.16 to −0.09)	0.88 (0.85 to 0.91)	**<0.001**
		IVW (fixed effects)	6	−0.11 (−0.14 to −0.08)	0.89 (0.87 to 0.92)	**<0.001**
		MR-PRESSO (Raw)	6	−0.09 (−0.16 to −0.02)	0.92 (0.85 to 0.98)	**0.04**
		MR-PRESSO (Outlier-corrected)	6	−0.06 (−0.10 to −0.03)	0.94 (0.91 to 0.97)	**0.02**
		MR-RAPS	6	−0.08 (−0.13 to −0.03)	0.92 (0.88 to 0.97)	**0.002**

*SNPs, Single nucleotide polymorphisms; OR, Odds ratio; CI, Confidence interval; IVW, inverse-variance weighted; 25(OH)D, 25-hydroxyvitamin D; HF, Heart failure*.

*Numbers in bold are statistically significant*.

### Sensitivity Analyses of MR

The leave-one-out method showed that a single SNP had little effect on the overall effect of causal correlation (Supplementary Figures 1, 2). MR-Egger regression intercept test and MR-PRESSO global test showed that there was no evidence of horizontal pleiotropy in either IVs of 25(OH)D or IVs of HF (All *P* > 0.05) (Table 4). However, in the heterogeneity test, the *p*-values of Cochran's Q statistics were all less than 0.05, indicating that there was heterogeneity between SNPs (Table 4). In order to deal with the influence of heterogeneity, we introduced the random-effect IVW method and the fixed-effect IVW method. The Random-effect IVW method was used as the gold standard in this study. The forest plot and funnel plot which could show the heterogeneity more intuitively were shown in Supplementary Figures 1, 2.

**Table 4:** Pleiotropy and heterogeneity test of the 25-hydroxyvitamin D genetic IVs from in Heart failure GWAS.

**Pleiotropy test**				**Heterogeneity test**					
**MR-egger**			**MR-PRESSO**	**MR-Egger**			**Inverse-variance weighted**		
**Intercept**	**SE**	***p***	***P***	**Q**	**Q_df**	**Q_*p*val**	**Q**	**Q_df**	**Q_*p*val**
0.002	0.003	0.50	0.13	218.62	135	<0.001	219.34	136	<0.001

## Discussion

No previous MR studies were conducted to investigate the causal association between vitamin D and heart failure. In this Mendelian randomization study, we observed evidence that genetically determined circulating 25(OH)D levels were associated with risk of heart failure. Furthermore, genetic susceptibility to heart failure was causally associated with decreased serum 25(OH)D levels. This was the first study with an adequate sample size under the assumption of Mendelian randomization to show a causal effect for a linear association between 25(OH)D concentrations and heart failure risk. Our results had potential significance for etiological understanding and disease prevention.

Vitamin D deficiency, one of the most common nutritional deficiencies, exists in all races and age groups, affecting more than 1 billion people worldwide (35). The overall evidence for the association between vitamin D and heart failure risk is mixed. A prospective study showed that the prevalence of heart failure increased with decreasing serum 25(OH)D levels (36). Lower serum 25(OH)D levels also tend to be associated with poor prognosis in heart failure (37, 38). At the same time, the level of serum 25(OH)D in patients with HF was often lower than that in the control group, so VD was also used as a serum marker to judge the prognosis of HF (39). Non-linear MR analysis showed that individuals with lower serum 25(OH)D levels were at increased risk of cardiovascular disease and elevated blood pressure, but analysis of cardiovascular disease subtypes was not performed (40). A meta-analysis indicated that vitamin D supplementation may prevent heart failure in older adults (41), which is consistent with our results. However, another meta-analysis of 21 studies indicated that vitamin D supplementation was not associated with heart failure mortality (42). One possible explanation is the interference of confounding factors to the research. Although the seasonal correlation of vitamin D levels was focused on by previous studies, seasonal changes in nitric oxide (NO) levels were often overlooked. As a classic hypotensive factor, NO diffuses rapidly in the blood after ultraviolet exposure (43), which is consistent with cardiovascular effects but not with serum 25(OH)D levels (44). Therefore, its protective effect on heart failure might be independent (44). Based on these findings, the 25(OH)D GWAS data were selected to exclude the interference of different seasons on the determination of vitamin D. At the same time, SNPs related to NO or NO production pathway were excluded on the basis of excluding traditional confounding factors of heart failure.

There were also differences in the results of previous randomized controlled trials. Several RCTs reports the same results. Positive effects of vitamin D supplementation on left ventricular structure and function (45, 46). However, other studies showed that vitamin D supplementation did not reduce mortality or improve left ventricular function (47–50). Our results challenged the interpretation of futility results from these randomized trials. One possible reason was that most previous studies on cardiovascular disease and mortality were conducted in people who did not lack vitamin D, so the possible benefits of vitamin D supplementation could not be ruled out.

Several potential mechanisms were proposed to explain the association of vitamin D with heart failure. Vitamin D deficiency may interfere with Ca^2+^ function in cardiomyocytes, consistent with an overload of Ca^2+^ in cardiomyocytes during HF (51). Low levels of vitamin D may activate the renin-angiotensin system and cause hyperparathyroidism (52). Vitamin D receptor is expressed in cardiomyocytes and vascular endothelial cells, which may be related to the regulation of vascular tension and atherosclerosis (53).

There are some strengthens in our study. Firstly, its design reduces potential confusion or reverse causality in observational studies. Secondly, our analysis also captures the risk of lifetime heart failure due to genetically reduced vitamin D, thus providing a stronger causal estimate than RCT. Thirdly, Reverse MR suggests that patients with heart failure tend to be more prone to vitamin D deficiency. Lastly, by using a two-sample MR method, we were able to test the role of vitamin D in a large group of patients with heart failure. This two-sample method has the same statistical power as the method of using individual level data (54).

However, our study also has certain limitations. Firstly, although the data sets of the two GWAS studies came from different sources, the GWAS data for heart failure included UKB. Although UKB is only one of 26 studies, this results in a small number of samples overlapped with the 25(OH)D dataset, which may cause minor deviations in the results. However, no other GWAS data that did not contain UKB data and were eligible are currently not available. Secondly, the potential pleiotropy could not be completely excluded, resulting in the three hypotheses cannot be accurately evaluated. However, we used a variety of methods for sensitivity analysis to get consistent results, which made the results of this study reassuring. Thirdly, all the subjects involved were of European ancestry, making the MR analysis unexplainable for the causal association between vitamin D and HF in other populations. In addition, IVs were drawn from summary-level data, so subgroup analyses were generally not possible. We also look forward to more detailed GWAS studies in the future.

## Conclusion

In summary, our MR analysis showed that there was a causal association between genetically predicted serum 25(OH)D levels and reduced risk of HF. In addition, the genetic susceptibility to HF was negatively correlated with the level of serum 25(OH)D. Considering the high mortality and huge disease burden of HF, low-cost, high-safety vitamin D supplementation has the potential to be a very promising treatment. Meanwhile, serum 25(OH)D level may be a potential molecular marker for the diagnosis and prognosis of HF. The results should be interpreted with caution, and further large-scale RCTs are warranted to validate our MR results.

## Data Availability Statement

The original contributions presented in the study are included in the article/Supplementary Materials, further inquiries can be directed to the corresponding authors.

## Author Contributions

NG and AD designed the study and drafted the article. DW, MN, and MK conducted data acquisition. NG, MK, MN, DW, ZH, XZ, YW, and AD performed data analysis and manuscript revision. All authors read and approved the final manuscript.

## Funding

This research was funded by Zhejiang Health Major Science and Technology Program, National Health Commission Scientific Research Fund (WKJ-ZJ-2121) and the National Natural Science Foundation of China (81800210).

## Conflict of Interest

The authors declare that the research was conducted in the absence of any commercial or financial relationships that could be construed as a potential conflict of interest.

## Publisher's Note

All claims expressed in this article are solely those of the authors and do not necessarily represent those of their affiliated organizations, or those of the publisher, the editors and the reviewers. Any product that may be evaluated in this article, or claim that may be made by its manufacturer, is not guaranteed or endorsed by the publisher.
